# Educational Applications of AI-Based Chatbots in Nursing: A Scoping Review

**DOI:** 10.3390/nursrep16030087

**Published:** 2026-03-03

**Authors:** Francisco Fernandes, Rúben Encarnação, José Alves, Carla Pais-Vieira, Suzinara Beatriz Soares de Lima, Paulo Alves

**Affiliations:** 1 Graduate Program in Nursing (PPGENF/UFSM), Federal University of Santa Maria, Santa Maria 97105-900, Brazil; suzinara.lima@ufsm.br; 2 Center for Interdisciplinary Research in Health (CIIS), Faculty of Health Sciences and Nursing, Catholic University of Portugal, 4169-005 Porto, Portugal; rcencarnacao@ucp.pt (R.E.); jcalves@ucp.pt (J.A.); cvieira@ucp.pt (C.P.-V.); pjalves@ucp.pt (P.A.)

**Keywords:** artificial intelligence governance, generative artificial intelligence, nursing education, large language models, educational technology, scoping review, clinical reasoning, ethics and governance

## Abstract

**Background/Objectives:** The rapid expansion of generative artificial intelligence (AI) and large language model-based chatbots has accelerated their adoption in higher education, including nursing. This scoping review mapped the use of AI-based chatbots in nursing education, including curricular domains, pedagogical approaches, educational outcomes, and implementation challenges. **Methods:** A scoping review was conducted following the Joanna Briggs Institute methodology and reported in accordance with the PRISMA-ScR guideline. Searches were performed across major bibliographic databases and grey literature sources. Quantitative, qualitative, and mixed-methods studies addressing the use of AI chatbots in nursing education or professional training were included. Data were extracted using a standardized instrument and synthesized through descriptive statistics and qualitative content analysis. **Results:** Sixty-six studies (2019–2025) were included, with significant growth observed after 2023. Most studies employed quasi-experimental designs (37.9%) and were implemented in academic settings (83.3%). Application formats varied across online, hybrid, simulation-based, and classroom models. Reported benefits included improved learning performance, clinical reasoning, and student engagement. Key challenges involved the reliability of AI outputs, academic integrity, data protection, and limited institutional governance. **Conclusions:** AI-based chatbots represent promising tools to enhance nursing education, particularly when integrated into structured pedagogical strategies with active faculty supervision. Their use can support the development of clinical reasoning, student engagement, and personalized learning. However, methodological heterogeneity, ethical concerns, and governance gaps highlight the need for careful implementation and further rigorous research to ensure safe, effective, and pedagogically sound integration.

## 1. Introduction

Artificial Intelligence (AI), a term established by John McCarthy in 1955, represents the capability of machines to perform tasks and solve problems that traditionally depend on human intelligence, such as natural language processing, pattern recognition, and decision-making [1,2,3]. Recently, AI has undergone significant evolution with the development of Generative Artificial Intelligence (GenAI). Powered by large language models (LLMs), GenAI can automatically generate diverse content, including text, images, audio, and video, by learning from large volumes of data [4,5]. Prominent examples such as ChatGPT, Google Gemini, and Llama illustrate this transformation, generating content that responds to specific user requests [6].

Among the most prominent AI tools, chatbots have been the focus of numerous studies in higher education, demonstrating the potential to positively impact various aspects of learning. Evidence suggests that incorporating these tools can lead to improved student outcomes, including greater satisfaction [7]. Within healthcare and health professions education, recent reviews indicate that integrating AI into curricula and professional education can contribute to enhanced learning, assessment, and competency development [8,9]. This digital and technological revolution requires educational programs and future professionals to adapt in order to keep pace with sectoral evolution and to be prepared to use AI ethically and effectively.

However, despite the increasing adoption of chatbots, there is a lack of structured evidence regarding their pedagogical design, educational effectiveness, and ethical considerations within nursing education, especially in light of recent advances in generative AI.

In the context of nursing, the adoption of new technologies in professional education is crucial for developing essential skills and competencies that meet real-world needs and emerging care models. Nursing education increasingly requires adaptability to innovations and the use of tools that simulate clinical scenarios, thereby enhancing decision-making and clinical reasoning. The World Health Organization (WHO) emphasizes the need for innovation in health education to strengthen the global workforce [10]. Given the growing integration of these technologies in healthcare and the potential demonstrated by chatbots in higher education, it becomes imperative to investigate how these AI tools can be incorporated and utilized to enhance nursing education.

Given the promise of improved education, a comprehensive mapping of the available evidence is warranted. Scoping reviews offer a robust methodological approach to examine the extent, scope, and nature of research activity on a given topic, as well as to identify knowledge gaps and research priorities [11].

A preliminary search of major databases (CINAHL, PubMed, Scopus, and Web of Science) and registries (Open Science Framework and PROSPERO) identified three reviews related to the use of chatbots in nursing education. The first, a systematic review by Zhang et al. [12], included only qualitative studies and conducted its search in November 2024, without incorporating grey literature. The second, a scoping review by Labrague and Sabei [7], similarly considered studies published up to 2024 but excluded grey literature sources and studies not published in English. The third, a scoping review protocol by Rodrigues et al. [13], while addressing Intelligent Tutoring Systems broadly, does not specifically focus on the distinct characteristics of conversational AI chatbots. Although these reviews provide valuable information, they do not offer a comprehensive and up-to-date mapping of the evidence, particularly regarding different study designs, emerging literature, and contributions from grey literature. Therefore, a new scoping review is needed to capture the full range of available evidence, including recent publications and grey literature, and to provide a more comprehensive understanding of how chatbots are being used in nursing education.

For the purposes of this review, AI-based chatbots were defined as computer-based conversational agents capable of interacting with users through natural language processing, including generative large language model-based systems, as well as rule-based or hybrid conversational agents used to support educational processes.

Given the above, it is pertinent to conduct a scoping review that investigates and maps the use of AI-based chatbots in nursing education, both in academic education and in the professional development of nurses and nursing students at undergraduate and postgraduate levels. Understanding the state of the art on this topic will help identify the potential, challenges, and gaps in the literature, thereby contributing to the advancement of pedagogical practices and technological innovation in nursing education.

Specifically, this review aims to:

- Identify the areas of the nursing curriculum in which chatbots are being applied.
- Describe how AI-based chatbots are being used, including the pedagogical strategies applied in nursing education.
- Map the main outcomes associated with the use of chatbots in nursing education.
- Identify the main challenges and limitations reported in integrating chatbots into nursing education.

## 2. Materials and Methods

This scoping review protocol was prospectively registered in the Open Science Framework (OSF) (DOI: 10.17605/OSF.IO/DBYA7) [14]. The review was conducted and reported in accordance with the PRISMA-ScR guideline. Given the emerging nature of the topic and the limited knowledge regarding the application of AI-based chatbots in nursing education, a scoping review was selected as the most appropriate methodological approach [11], as it allows for the comprehensive mapping of available evidence irrespective of study design.

The review followed the methodology recommended by the Joanna Briggs Institute (JBI) for scoping reviews [11,15]. Following protocol registration, the manuscript title was refined to improve clarity and alignment with the final scope of the review. This modification was limited to the title and did not affect the research objectives, eligibility criteria, methodological approach, or analytical framework defined in the original protocol.

The review followed systematic steps, including the formulation of the research question, comprehensive literature searching, screening of eligible studies, data extraction and organization, evidence synthesis, and structured presentation of the results. The completed PRISMA-ScR checklist [16] is provided as Supplementary Material (Table S1).

### 2.1. Research Question

In scoping reviews, it is recommended that research questions be formulated broadly and clearly to encompass the concept to be explored, the target population, and the outcomes or context of interest, thereby guiding a systematic and comprehensive search [17].

To achieve the study objectives, the research question was formulated using the PCC mnemonic (Population, Concept, Context): What evidence currently exists regarding the use of AI-based chatbots in nursing education?

### 2.2. Search Strategy

To ensure comprehensive coverage of the available literature, systematic searches were conducted across multiple electronic databases, including PubMed, CINAHL Complete, Scopus, Web of Science, SciELO, Cochrane Library, and VHL/LILACS. In addition to bibliographic databases, grey literature sources were searched through OpenAIRE, Open Dissertations, BDTD/CAPES, ProQuest™ Dissertations & Theses Citation Index, and Google Scholar in order to identify relevant materials not indexed in conventional journals. This combined strategy was designed to maximize sensitivity and ensure broad identification of evidence on AI-based chatbots in nursing education.

The final searches across all sources, including bibliographic databases and grey literature, were completed on 13 October 2025. This date was considered the definitive search date for the purposes of this review. The complete search strategies for each database are provided in Table S2 in the Supplementary Material.

The Google Scholar search was performed using a structured query with the restrictive operator allintitle in order to increase retrieval specificity and prioritize studies explicitly focused on AI-based chatbots in nursing education. This approach was intentionally adopted to enhance alignment with the scope of the review and reduce the retrieval of irrelevant records, which is consistent with recommended practices for improving precision in Google Scholar searches on emerging topics.

All records retrieved from Google Scholar were saved within the platform and subsequently exported using the built-in citation export function in RefMan (RIS) format. These exported records were then imported into the Rayyan web platform (Rayyan Systems Inc., Cambridge, MA, USA), available at https://www.rayyan.ai (accessed on 20 October 2025) [18], where they were combined with records retrieved from other grey literature sources and bibliographic databases.

### 2.3. Eligibility Criteria

Eligibility criteria were defined using the JBI PCC framework. The Population comprised nursing students and professionals; the Concept focused on AI-based chatbots as educational tools; and the Context included teaching–learning processes and professional training in nursing.

Studies were included if they analyzed the use of AI-based chatbots in formal educational contexts, such as undergraduate and postgraduate programs, as well as in non-formal contexts, including training courses, continuing education, and professional development programs. Research focused exclusively on AI applications in clinical care, management, or diagnostic contexts, without a direct relationship to teaching–learning processes, was excluded.

To ensure a comprehensive mapping of the literature, this review included a wide range of empirical evidence, such as quantitative, qualitative, and mixed-methods studies encompassing experimental, quasi-experimental, cross-sectional, developmental, implementation, and case study designs, as well as grey literature sources reporting original empirical data from theses and dissertations. Only studies that explicitly addressed the use of AI-based chatbots in teaching, learning, or professional development within nursing education contexts were considered. Secondary research articles (e.g., systematic or narrative reviews), conceptual or theoretical articles, expert commentaries, discussion papers, consensus documents, educational reference materials, editorials, and letters to the editor were excluded.

The registered protocol initially allowed the inclusion of review studies; however, during the review process, the eligibility criteria were refined to include only primary studies in order to directly map original evidence and avoid duplication of synthesized findings. This modification did not affect the review objectives or overall methodological approach. Protocol deviations were transparently reported in accordance with PRISMA-ScR recommendations [11,16], ensuring methodological transparency and consistency.

Sources published in any language and from any year were considered, aiming to provide a complete mapping of relevant evidence. The review team possesses proficiency in English, Spanish, and Portuguese, allowing direct evaluation of studies published in these languages. For articles published in other languages, translations were arranged as needed to reduce language bias and ensure inclusion.

### 2.4. Evidence Screening and Study Selection

The study selection process was conducted in structured and sequential phases to ensure methodological rigor and transparency. Prior to full screening, a calibration exercise was performed to refine the application of the eligibility criteria and align reviewers’ interpretations.

Following the execution of the search strategies, all retrieved records were imported into the Rayyan web platform [18]. Duplicate records were automatically identified by the platform and subsequently verified and removed manually by the reviewers. The remaining records underwent independent title and abstract screening according to the predefined eligibility criteria.

Records considered potentially eligible were exported to Zotero software (v8.0.3; Corporation for Digital Scholarship, Vienna, VA, USA), which was used to retrieve, manage, and organize full-text reports for detailed assessment. No ar-bitrary numerical limits were applied to the Google Scholar search, and all retrieved records were screened.

Full-text reports were obtained through institutional access or, when necessary, by contacting the corresponding authors. Two reviewers independently assessed each full-text report based on the predefined PCC criteria. Discrepancies at any stage were resolved through discussion and consensus, with consultation of a third reviewer when required to ensure consistency in the final decision. Reference lists of included studies were also manually screened to identify additional relevant publications.

Inter-rater agreement for full-text eligibility assessment was calculated using Cohen’s kappa coefficient. Agreement was substantial (κ = 0.82), indicating high consistency between reviewers prior to consensus resolution.

### 2.5. Data Extraction and Organization

Data extraction was conducted using a structured spreadsheet developed specifically for this review to ensure consistency and transparency. The extracted variables included publication year, country of origin, study design, characteristics of the AI-based chatbot (e.g., rule-based or generative), educational context, target population, implementation setting, pedagogical strategy, and reported educational outcomes.

The classification of chatbot types, educational applications, pedagogical strategies, and outcomes followed a combined inductive and deductive approach. Initial categories were informed by existing educational and technological frameworks and were iteratively refined during the data extraction process to reflect patterns observed across the included studies. The categories were not mutually exclusive, as chatbot implementations frequently encompassed multiple functions and educational purposes.

Explicit decision rules were applied to guide category assignment. Studies were classified into all relevant categories when sufficient information was provided in the methods, intervention description, or results sections. No forced single-category assignment was applied. For example, when a chatbot was used both as a learning support tool and as a virtual tutor, the study was assigned to both categories. When classification information was unclear or insufficient, categorization was based solely on explicitly reported data, and no assumptions were made.

Data extraction and categorization were performed independently by two reviewers. Discrepancies were resolved through discussion and consensus to ensure consistency and methodological rigor. A descriptive synthesis was subsequently undertaken to summarize study characteristics, technological approaches, and educational implementation trends, consistent with the objectives of a scoping review.

Data extraction was conducted using a structured spreadsheet developed specifically for this review to ensure consistency and transparency. The extracted variables included publication year, country of origin, study design, characteristics of the AI-based chatbot (e.g., rule-based or generative), educational context, target population, implementation setting, pedagogical strategy, and reported educational outcomes.

### 2.6. Data Analysis and Synthesis

Data analysis was conducted by the reviewers involved in the previous stages, following an approach compatible with the objectives of a scoping review. Quantitative data were analyzed using descriptive statistics, while qualitative data were synthesized using content analysis.

Included publications were grouped into analytical categories according to how AI was applied to support nursing education. Considering the methodological, conceptual, and outcome diversity of the included studies, meta-analysis was not feasible, which is consistent with the exploratory and descriptive nature of this type of review [11].

The results are presented using a structured descriptive approach supported by summary tables and figures. This format was selected to improve readability and facilitate synthesis, given the heterogeneity of study designs, chatbot technologies, educational settings, and reported outcomes.

## 3. Results

The results are presented using a structured descriptive approach supported by summary tables and figures. This format was selected to improve readability and support synthesis, given the heterogeneity of study designs, chatbot technologies, educational settings, and outcomes.

### 3.1. Study Selection

The database searches identified 2957 records. After removing 1364 duplicate records, 1593 records were screened by title and abstract, of which 1281 were excluded. A total of 312 reports were sought for retrieval and assessed for full-text eligibility, of which 262 were excluded for not meeting the eligibility criteria, resulting in the inclusion of 50 studies from bibliographic databases. The reasons for exclusion at the full-text stage were systematically recorded and categorized in accordance with the predefined PCC eligibility framework. The most frequent reasons included the absence of a direct focus on nursing education contexts, lack of implementation of chatbot-based AI interventions, or the presentation of secondary or non-empirical publications. Additional exclusions involved studies primarily addressing clinical applications without an explicit educational component, protocol-only reports without results, and publications with insufficient methodological detail to allow reliable categorization. These decisions were applied consistently across reviewers following independent assessment and consensus procedures to ensure methodological coherence and transparency.

In addition, 1014 records were identified through other sources, including grey literature searches. After screening 891 records by title and abstract, 842 were excluded. The remaining 49 reports were assessed for full-text eligibility, and 33 were excluded, resulting in the inclusion of 16 studies from grey literature sources.

**Figure 1:**
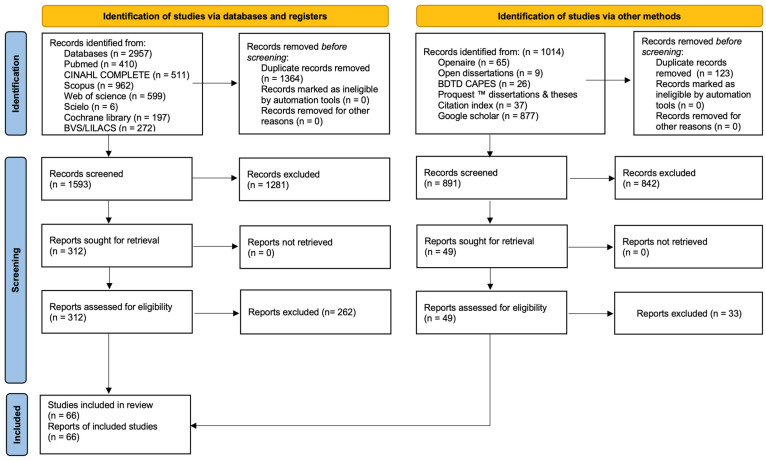
PRISMA 2020 flow diagram [16].

Overall, 66 studies met all eligibility criteria and were included in the final synthesis [19,20,21,22,23,24,25,26,27,28,29,30,31,32,33,34,35,36,37,38,39,40,41,42,43,44,45,46,47,48,49,50,51,52,53,54,55,56,57,58,59,60,61,62,63,64,65,66,67,68,69,70,71,72,73,74,75,76,77,78,79,80,81,82,83,84]. The study selection process is presented in Figure 1, in accordance with the PRISMA-ScR guidelines [15].

A detailed numerical reconciliation of the study selection process, including records identified, duplicates removed, screened records, full-text assessments, and final inclusions across bibliographic databases and grey literature sources, is provided in Supplementary Material (Table S4). In addition, Table S4 presents the categorized reasons for full-text exclusion to ensure full methodological transparency and auditability of the selection process, in accordance with PRISMA 2020 reporting recommendations.

### 3.2. Study Characteristics

The characteristics of the included publications are summarized in Table 1, with the full results presented in the corresponding data extraction table (see Supplementary Material, Table S3).

The 66 included studies were published between 2019 and 2025, with a marked increase from 2023 onward. Most publications occurred in 2025 (n = 43; 65.2%) [19,20,21], followed by 2024 (n = 12; 18.2%) [22,23] and 2023 (n = 6; 9.1%) [24,25]. Earlier years contributed only a small number of studies, including three publications in 2022 [26,27] and isolated studies published in 2021 and 2019 [28,29], reflecting the recent and rapidly expanding nature of research on AI-based chatbots in nursing education, reflecting the recent and rapidly expanding nature of research on AI-based chatbots in nursing education. Of the 66 included studies, five were preprints that had not yet undergone peer review at the time of data extraction. These were retained to ensure comprehensive mapping of this rapidly evolving field.

Geographically, research activity was concentrated in Asia (n = 42; 63.6%) [30], particularly in China [31], Taiwan [32,33], and South Korea [19,30]. North America contributed seven studies, all from the United States [34,35], while Europe [26,36], Africa, South America, and Oceania were less represented [37]. A small proportion of studies involved international collaborations [38,39], indicating emerging global engagement but an uneven regional distribution.

Methodologically, quasi-experimental designs predominated (n = 25; 37.9%) [23,27,40], followed by qualitative and cross-sectional approaches. Randomized controlled trials were comparatively scarce, and several studies adopted developmental, methodological, or quality improvement designs. Most investigations targeted undergraduate nursing students, with fewer studies addressing postgraduate or continuing professional education. In terms of educational implementation, chatbots were primarily used to support learning-focused activities, with a smaller number integrating teaching and assessment functions.

Implementation settings were predominantly academic (n = 55; 83.3%) [23,27,41], and delivery formats varied across online, blended, simulation-based, and classroom-based models. Intervention duration ranged from single-session applications to multi-week or course-embedded designs, although reporting of duration was inconsistent across some studies.

### 3.3. Technological and Educational Applications of AI Chatbots

Across the included studies, large language model (LLM)-based generative systems predominated [42,43], with ChatGPT representing the most frequently reported tool [23,27,44]. Additional generative AI applications were described in a smaller subset of studies [45,46], while earlier implementations more commonly relied on rule-based or knowledge-based architectures [25,28]. A limited number of studies employed adaptive non-generative systems or AI-driven virtual patient simulations [33], reflecting technological heterogeneity across publication years.

Chatbots were primarily deployed through web-based interfaces, including direct access to LLM platforms and web-integrated educational systems [23,27,44], with fewer implementations embedded in mobile applications, institutional learning management systems, or clinical simulation environments [28,33,40]. This distribution underscores the rapid adoption of accessible generative AI platforms within higher education settings.

From a pedagogical perspective, AI-based chatbots were most frequently positioned as supplementary learning support tools. Common applications included self-directed study assistance, clarification of academic content, scaffolded tutoring, and guided feedback [23,27,41]. More advanced integrations involved clinical case discussions, scenario-based reasoning, and virtual patient simulations [28,33], targeting higher-order competencies such as clinical judgment, communication skills, and decision-making.

Curricular integration spanned foundational nursing knowledge, specialty areas, and simulation-based learning contexts. Improvements in knowledge acquisition and short-term academic performance were the most consistently reported outcomes [23,27,40], while gains in clinical reasoning were more frequently associated with case-oriented and simulation-based applications [28,33]. At the affective level, increased engagement, motivation, and perceived usefulness were recurrent findings [23,41,45], although concerns regarding reliability, trust in AI-generated outputs, and academic integrity were also reported [44,47].

Overall, AI-based chatbots have largely been adopted as complementary tools within existing curricular structures rather than as fully integrated pedagogical systems. Learning support and tutoring remain the dominant applications, whereas simulation-based and reasoning-oriented implementations appear more closely aligned with the development of advanced clinical competencies.

### 3.4. Educational Applications and Outcomes

The included studies reported the application of AI-based chatbots across multiple domains of nursing education, frequently addressing more than one curricular area within the same intervention. Chatbots were most commonly applied to general nursing knowledge and core curriculum topics (n = 15; 22.7%) [23,27], followed by applications targeting clinical reasoning and the nursing process (n = 10; 15.2%) [33,40], as well as clinical simulation and case-based learning (n = 9; 13.6%) [28,33]. Additional applications encompassed specialty nursing areas such as pediatrics, mental health, and critical care (n = 7; 10.6%) [23,41]; maternal and obstetric nursing (n = 6; 9.1%) [33]; communication skills and clinical history-taking (n = 6; 9.1%) [25,28]; and academic writing and research skills (n = 6; 9.1%) [44,45]. Less frequently, chatbots supported medical terminology acquisition (n = 4; 6.1%) [27] and educational technology or AI ethics content (n = 3; 4.5%) [47].

Pedagogically, integration strategies were predominantly pragmatic and functional. Most studies embedded chatbots as supportive tools within existing teaching and learning processes rather than as components of formally articulated educational frameworks [23,27,44]. Learning-centered strategies predominated, particularly self-directed study support, clarification of doubts, content reinforcement, and the provision of immediate feedback [23,27,41]. A subset of studies incorporated chatbots into virtual tutoring, guided case discussions, and formative assessment activities [44,45], while more advanced implementations involved clinical case simulations and virtual patient scenarios designed to strengthen clinical reasoning and decision-making competencies [28,33]. Although these approaches align conceptually with case-based and simulation-based pedagogies, explicit theoretical frameworks were rarely reported.

Reported outcomes spanned cognitive, affective, and behavioral domains [23,27]. Improvements in knowledge acquisition and learning performance were the most frequently documented outcomes (n = 18) [23,27,44], particularly in quasi-experimental and controlled studies. Gains in skills and competency development (n = 14) [33,40], as well as improvements in clinical reasoning and critical thinking (n = 9), were more commonly associated with simulation-based and case-oriented applications [28,33]. At the affective level, chatbot use was associated with increased engagement, motivation, and self-directed learning (n = 9) [23,41], along with positive perceptions of usefulness and accessibility (n = 8) [44,45]. However, variability in trust toward AI-generated outputs and concerns regarding reliability and academic integrity were also reported [44,47].

Table 2 summarizes the main application areas and their associated educational outcomes.

Educational domains were coded as non-mutually exclusive categories; therefore, individual studies could contribute to more than one application area.

**Figure 2:**
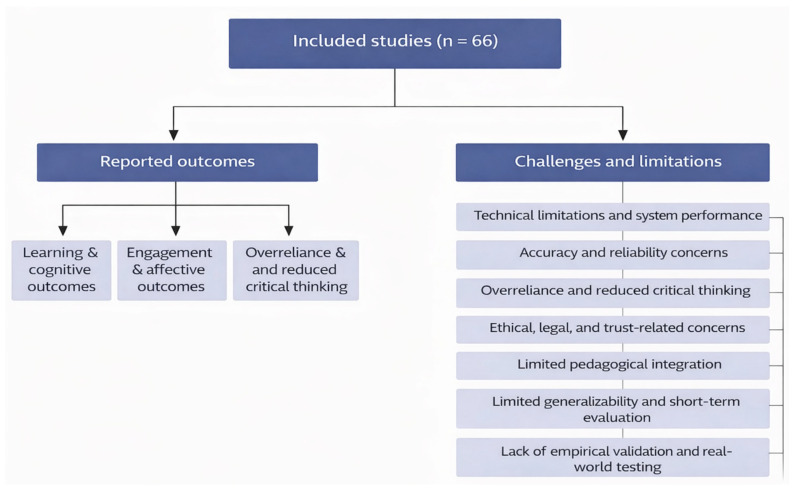
Synthesis of reported outcomes and challenges identified in studies on AI-based chatbots in nursing education.

Figure 2 presents a conceptual synthesis integrating the reported educational benefits and implementation challenges identified across the included studies.

### 3.5. Implementation Challenges and Barriers

The integration of AI-based chatbots into nursing education revealed recurring challenges across technological, pedagogical, ethical, and organizational dimensions [44,47]. Technical limitations included restricted functionality, system instability, and the need for ongoing technical support during implementation [27,45,46]. Concerns regarding the accuracy and reliability of AI-generated information were frequently emphasized, particularly given the implications for patient safety in health education contexts [25,44,47]. Several studies highlighted the importance of continuous content validation and expert supervision to ensure safe and pedagogically appropriate use [23,40].

Pedagogical challenges were often linked to limited curricular integration and insufficient educator preparation. The absence of clearly articulated instructional frameworks contributed to superficial or supplementary adoption rather than systematic integration [23,27,40]. Additionally, concerns about potential overreliance on chatbots and reductions in independent critical thinking were reported, particularly when chatbot use occurred without structured pedagogical guidance [41,44,45]. Discussions surrounding academic integrity, authorship, and appropriate use in assessment contexts were also identified [44,47].

Ethical and legal considerations—including data privacy, confidentiality, and trust in AI-generated responses—were reported across several studies [25,47]. Many interventions were conducted within single institutions, involved small sample sizes, or had short durations, thereby limiting generalizability and long-term inferences regarding educational impact [33,40,41]. Furthermore, some chatbot applications remained at pilot or early implementation stages, lacking robust empirical validation or real-world testing within nursing education programs [28,46].

## 4. Discussion

This scoping review mapped 66 studies on the use of AI-based chatbots and related systems in nursing education, revealing a marked increase in publications from 2023 onward and a strong concentration in recent years. This temporal pattern reflects the rapid diffusion of generative AI in academic environments and its pragmatic incorporation into educational practice, largely driven by the accessibility of large language model-based tools such as ChatGPT. Similar trends have been observed across broader educational contexts, where the adoption of generative AI has accelerated pedagogical experimentation and research production [23,31].

Overall, the included studies consistently reported educational benefits, particularly improvements in knowledge acquisition, academic performance, skills development, and clinical reasoning. These outcomes were more frequently demonstrated in quasi-experimental and controlled studies, which reported measurable gains in learning performance and simulated clinical tasks, while qualitative and cross-sectional studies provided complementary insights into student engagement, acceptance, and perceived usefulness [19,20,21,22]. Collectively, these findings support the role of AI-based chatbots as effective supplementary tools within the nursing teaching–learning process, particularly when aligned with pedagogical objectives and intentionally integrated into instructional design.

Most implementations positioned chatbots as auxiliary resources to support studying, clarify doubts, and assist with academic tasks, contributing to increased learner autonomy, efficiency, and motivation [23,30,37]. This pattern of use may enable educators to devote greater attention to higher-order pedagogical activities, including clinical discussion, reflective supervision, and formative assessment, thereby reinforcing the central role of faculty mediation. More advanced applications, such as virtual tutors, AI-generated clinical cases, and virtual patient simulations, although less frequently reported, were more directly associated with the development of applied competencies, including clinical communication, decision-making, and diagnostic reasoning [26,28,35]. These findings highlight that the educational value of chatbots depends not solely on technological capability but on their integration within structured pedagogical strategies and supervised learning environments.

The integration of chatbots into simulation-based learning environments further demonstrated the potential to enhance realism, interactivity, and individualized feedback, particularly during structured phases such as clinical case analysis and guided reflection. These findings align with established simulation-based learning literature, which emphasizes the importance of instructional structure and guided debriefing in promoting clinical competence development [85,86]. However, concerns related to response accuracy, clinical realism, and the need for expert validation indicate that AI should be implemented as a supportive component within instructional design rather than as a replacement for human facilitation [19,33].

From a theoretical perspective, these findings can be interpreted through constructivist learning theory and self-regulated learning models, in which learners develop knowledge through guided interaction, feedback, and reflection [87]. AI-based chatbots may contribute to these processes by providing accessible, immediate, and adaptive feedback, thereby supporting metacognitive engagement and autonomous learning.

A cross-cutting finding identified across the included studies relates to the performance–trust paradox. Although chatbots demonstrated adequate performance in specific educational tasks, students and educators frequently reported lower levels of trust in AI-generated outputs [22]. This discrepancy reflects broader challenges in human–AI interaction, where perceived reliability, transparency, and explainability influence trust calibration and user acceptance [88]. In nursing education, these findings emphasize the importance of ensuring content accuracy, promoting critical appraisal skills, and maintaining appropriate pedagogical supervision, particularly in contexts involving clinical reasoning.

Ethical and academic integrity considerations also emerged as central themes. Studies reported concerns related to plagiarism, overreliance on AI tools, unclear authorship attribution, and the absence of consistent institutional policies [44,47,48]. Existing literature suggests that effective management of these risks requires comprehensive institutional strategies, including clear guidelines, transparent disclosure of AI use, and assessment designs aligned with authentic competencies and critical reasoning [89]. These considerations are particularly relevant in nursing education, where professional responsibility, ethical conduct, and patient safety constitute core educational outcomes.

Beyond pedagogical considerations, the integration of AI-based chatbots in nursing education requires alignment with institutional governance and data protection frameworks. Regulatory instruments such as the General Data Protection Regulation (GDPR) and the European Union Artificial Intelligence Act emphasize transparency, accountability, and human oversight in AI deployment [90,91]. These frameworks highlight the importance of ensuring lawful, responsible, and ethically grounded implementation of AI technologies, particularly in domains closely linked to clinical practice and public trust [92,93].

Taken together, the findings of this scoping review indicate that AI-based chatbots have substantial potential to support nursing education when integrated as complementary tools within structured pedagogical frameworks. Their educational value depends not only on technological capabilities but also on appropriate instructional design, faculty supervision, and institutional governance, reinforcing the importance of aligning technological innovation with established educational principles.

### 4.1. Practical Implications and Challenges

The integration of chatbots and large language models into nursing education has occurred predominantly through the pragmatic adoption of readily available tools, often preceding formal curricular integration. Evidence suggests that educational outcomes are more favorable when chatbots are used as supplementary tools rather than as replacements for teaching, particularly when accompanied by active faculty supervision, especially in activities involving clinical reasoning and decision-making. This approach aligns with constructivist learning models that emphasize guided autonomy and metacognitive development [87].

A major challenge concerns the reliability and safety of AI-generated outputs. Although chatbots have demonstrated adequate performance in specific tasks, variability in response accuracy and perceived reliability highlights the need for verification protocols, clear communication of AI limitations, and the development of learners’ critical appraisal skills. Aligning AI-generated content with clinical guidelines, institutional protocols, and evidence-based standards is essential to ensure pedagogical validity and patient safety.

Ethical and academic integrity considerations also require structured institutional responses. The widespread availability of AI tools necessitates clear policies regarding acceptable use, transparent disclosure, and assessment strategies that prioritize reasoning, clinical judgment, and reflective practice [89]. Additionally, data protection, privacy, and governance considerations must be addressed, particularly when external platforms are used. Ensuring compliance with regulatory requirements and establishing institutional oversight mechanisms are essential to support the responsible and ethical implementation of AI technologies in nursing education.

Educators should integrate AI-based chatbots through structured instructional design aligned with defined learning objectives and clinical competencies. Faculty supervision, clear pedagogical framing, and appropriate integration into teaching strategies are essential to maximize educational benefits while mitigating potential risks.

### 4.2. Future Directions

Despite the rapid growth of evidence in this field, several gaps remain and should guide future research. First, the predominance of quasi-experimental designs and self-reported outcomes highlights the need for multicenter studies with longitudinal follow-up and the use of objective performance measures, such as OSCE stations, standardized rubrics, and simulation-based assessments. Anchoring evaluations in established educational evaluation models may help distinguish short-term learning gains from sustained changes in professional performance [94].

Second, the adoption and implementation of AI-based chatbots should be examined through the lens of theoretical models of technology acceptance and use. Frameworks such as the Technology Acceptance Model (TAM), the Theory of Planned Behavior (TPB), and the Unified Theory of Acceptance and Use of Technology (UTAUT) can help elucidate determinants related to perceived usefulness, ease of use, social norms, and behavioral intentions [95,96,97,98]. These models may be complemented by implementation science frameworks, such as the Consolidated Framework for Implementation Research (CFIR), to identify organizational barriers and facilitators and to support the sustainability of educational interventions involving AI [99].

Third, future studies should systematically compare different technological and pedagogical approaches, including rule-based chatbots versus LLM-based systems, prompting strategies, integration with simulation and virtual reality, and differential effects across educational levels (undergraduate, postgraduate, and continuing professional education). Greater alignment with emerging reporting and validation guidelines for AI-based interventions, such as CONSORT-AI and SPIRIT-AI, is also recommended to enhance transparency, reproducibility, and methodological rigor [100].

Finally, future research should explicitly address issues of equity, accessibility, and contextualization, including linguistic diversity, digital access, institutional resources, and cultural appropriateness, in order to prevent the integration of generative AI from exacerbating existing educational inequalities.

### 4.3. Implications for Clinical Practice Readiness

Beyond their immediate educational applications, AI-based chatbots may also contribute to the development of clinical readiness among nursing students. Evidence from the included studies suggests that chatbot-supported learning environments can foster clinical reasoning through scenario-based problem solving and structured decision-making exercises. For example, one study demonstrated improvements in students’ ability to interpret clinical situations and prioritize nursing actions within simulated contexts [30]. Similarly, another study highlighted the potential of AI-driven chatbot interactions to support reflective clinical judgment and guided reasoning processes [40].

In addition to supporting decision-making skills, AI-based chatbots may serve as tools for developing communication and patient education competencies. Simulated dialogue with virtual patients has been shown to allow learners to practice explaining health-related information and adapting communication strategies to diverse scenarios [33]. Such applications align with competency-based nursing education approaches that emphasize not only knowledge acquisition but also critical thinking, communication, and professional preparedness.

However, it is important to distinguish between the pedagogical use of chatbots as learning support tools and their potential deployment as clinical decision-support systems. Although several studies reported increased engagement, confidence, and perceived competence, many relied on short-term or self-reported outcomes. Therefore, further longitudinal and performance-based research is needed to determine whether chatbot-assisted education translates into measurable improvements in real-world clinical practice.

### 4.4. Limitations

The limitations of this review operate at two interrelated levels.

First, limitations inherent to the included evidence must be acknowledged. Many studies were conducted within single institutions or specific educational contexts, frequently involved small sample sizes, had short intervention durations, and relied heavily on self-reported outcomes. The substantial methodological and conceptual heterogeneity across study designs, technological architectures, pedagogical strategies, and outcome measures limited direct comparability and precluded conclusions regarding sustained or transferable effects on clinical competence development. In addition, the use of the allintitle operator in Google Scholar, while increasing search specificity, may have reduced sensitivity and resulted in the omission of potentially relevant studies.

Second, as characteristic of scoping review methodology, the primary aim of this study was to map the breadth, nature, and distribution of evidence rather than to evaluate effectiveness through quantitative synthesis or causal inference. Accordingly, no formal critical appraisal of methodological quality or risk of bias was undertaken, consistent with established methodological guidance for scoping reviews [11,17,66,101]. While this approach prioritizes comprehensive coverage, it limits the ability to assess the internal validity of individual studies or to weigh findings according to methodological rigor.

The inclusion of preprint studies represents an additional consideration. Although these reports had not undergone formal peer review at the time of data extraction, they were retained to ensure comprehensive coverage of this rapidly evolving field. Importantly, a sensitivity analysis excluding preprints did not materially alter the overall thematic distribution of technological applications, pedagogical strategies, or reported outcomes, suggesting that their inclusion did not substantively influence the principal conclusions.

The rapid evolution of generative artificial intelligence technologies introduces a structural risk of partial obsolescence. New models, deployment frameworks, governance regulations, and empirical findings continue to emerge at an accelerated pace. Consequently, periodic updates and future systematic reviews incorporating formal quality appraisal and longitudinal outcome assessment will be necessary to consolidate and extend the present findings. Furthermore, publication bias and selective reporting cannot be excluded, particularly given the novelty and positive framing frequently associated with generative AI innovations.

## 5. Conclusions

This scoping review provides a comprehensive and up-to-date mapping of the rapidly expanding integration of AI-based chatbots in nursing education, capturing the post-2023 acceleration driven by generative large language model technologies. Beyond documenting growth trends, the review synthesizes how technological architectures, pedagogical strategies, and governance considerations intersect within nursing education contexts.

Across diverse settings, AI-based chatbots have primarily functioned as pedagogically supportive tools rather than autonomous instructional systems. Their educational contribution appears most meaningful when embedded within structured learning designs, aligned with curricular objectives, and mediated through active faculty supervision. Applications involving simulation, clinical reasoning, and guided tutoring demonstrate particular promise for fostering higher-order competencies, although their effectiveness remains contingent upon instructional coherence and contextual integration.

At the same time, substantial methodological heterogeneity, limited theoretical grounding, and the predominance of short-term and self-reported outcomes constrain the strength of current inferences. Ethical, governance, and accountability challenges—particularly those related to trust calibration, academic integrity, data protection, and institutional oversight—emerge as central determinants of responsible implementation.

Collectively, the evidence suggests that the educational value of AI-based chatbots in nursing does not reside solely in technological capability but in their integration within ethically governed, pedagogically intentional, and institutionally supported frameworks. Future research should advance beyond exploratory designs toward longitudinal, multicenter, and performance-based evaluations aligned with standardized reporting and regulatory guidance.

By consolidating dispersed evidence and highlighting structural, pedagogical, and governance dimensions, this review contributes a foundation for more theoretically grounded and policy-informed integration of AI technologies in nursing education. Ensuring that AI adoption strengthens professional standards, safeguards patient safety, and promotes equitable access will be essential for translating innovation into sustainable educational advancement.

## Figures and Tables

**Table 1:** Study characteristics (n = 66).

	n	%
Year of Publication		
2019	1	1.5
2021	1	1.5
2022	3	4.5
2023	6	9.1
2024	12	18.2
2025	43	65.2
Region		
Europe	3	4.5
North America	7	10.6
Asia	42	63.6
Africa	5	7.6
South America	3	4.5
Oceania	1	1.5
Multiple regions	5	7.6
Study design		
Quasi-experimental study	25	37.9
Randomized controlled trial	4	6.0
Cross-sectional surveys	8	12.1
Qualitative study	14	21.2
Mixed-methods study	7	10.6
Methodological/developmental study	4	6.0
Case studies/quality improvement initiatives	4	6.0

Note: Percentages were calculated based on the total number of included studies (n = 66) and rounded to one decimal place.

**Table 2:** Summary of chatbot application areas in nursing education and the most frequently reported outcomes.

Application Area	Number of Studies	Examples of Use	Most Frequently Reported Outcomes
Learning support	34	Self-directed study, clarification of doubts, academic task assistance, concept explanation	Improved knowledge acquisition, increased autonomy, enhanced engagement, improved academic performance
Clinical simulation	11	Virtual patients, case-based interaction, scenario-based reasoning, simulation support	Improved clinical reasoning, increased confidence, enhanced decision-making skills
Virtual tutoring	12	Guided feedback, question-answer interaction, personalized tutoring, scaffolding	Improved learning outcomes, increased engagement, improved skill acquisition
Teaching support	6	Content preparation, instructional material generation, teaching assistance	Increased teaching efficiency, improved instructional design
Assessment and skills practice	9	Formative quizzes, structured clinical responses, skills rehearsal, competency assessment	Improved skill performance, reinforcement of learning, enhanced competency development

## Data Availability

All data supporting the findings of this study are available through open-access repositories. The review protocol, search strategies, and Supplementary Materials are published in the Athena Health & Research and registered in the Open Science Framework (OSF). The published protocol is accessible at https://doi.org/10.62741/ahrj.v3iSuppl.124, and the OSF registry can be found at https://doi.org/10.17605/OSF.IO/DBYA7. No new primary data were generated for this scoping review.

## Supplementary Materials

The following supporting information can be downloaded at: https://www.mdpi.com/article/10.3390/nursrep16030087/s1, Table S1: PRISMA-ScR Checklist; Table S2: Search Strategy; Table S3: Extracted Data; Table S4: Numerical Reconciliation of Study Selection Process and Reasons for Full-Text Exclusion. Ref. [102] is cited in the Supplementary Materials file.

## Public Involvement Statement

No public involvement in any aspect of this research.

## Guidelines and Standards Statement

This scoping review was conducted in accordance with the JBI methodology for scoping reviews [11], and this manuscript was drafted against the Preferred Reporting Items for Systematic Reviews and Meta-Analyses extension for Scoping Reviews (PRISMA-ScR) reporting guideline [16].
