# Supplementary material for: Educational Applications of AI-Based Chatbots in Nursing: A Scoping Review
Source: Nurs Rep. 2026 Mar 3;16(3):87. doi: 10.3390/nursrep16030087 (PMC13028657; doi:10.3390/nursrep16030087)
Supplement: Supplementary file 1 [file nursrep-16-00087-s001.zip › nursrep-4153709-Table S2.pdf]

Table S2. Search Strategy.

Database	Search strategy	Results
MEDLINE via PUBMED	"nurs*"[Title/Abstract] OR "nursing student*"[Title/Abstract] OR "undergraduate nursing"[Title/Abstract] OR "Students, Nursing"[MeSH Terms] OR "nurses"[MeSH Terms] OR "Nursing Staff"[MeSH Terms] AND "chatgpt"[Title/Abstract] OR "chat gpt"[Title/Abstract] OR "natural language processing"[Title/Abstract] OR "chatbot*"[Title/Abstract] OR "conversational agent*"[Title/Abstract] OR "artificial intelligent agent*"[Title/Abstract] OR "artificial intelligence agent*"[Title/Abstract] OR "intelligent agent*"[Title/Abstract] OR "AI agent"[Title/Abstract] OR "ai based conversational agent*"[Title/Abstract] OR "intelligent conversational agent*"[Title/Abstract] OR "artificial agent*"[Title/Abstract] OR "virtual assistant*"[Title/Abstract] OR "conversational agent*"[Title/Abstract] OR "ai tutor*"[Title/Abstract] OR "intelligent tutor*"[Title/Abstract] OR "automated tutor*"[Title/Abstract] OR "virtual agent*"[Title/Abstract] OR "intelligent virtual assistant*"[Title/Abstract] OR "natural language processing"[MeSH Terms] OR "Generative Artificial Intelligence"[MeSH Terms] AND educat*"[Title/Abstract] OR "teach*"[Title/Abstract] OR "training"[Title/Abstract] OR "instruction*"[Title/Abstract] OR "simulat*"[Title/Abstract] OR "interactive learning"[Title/Abstract] OR "gamification"[Title/Abstract] OR "game*"[Title/Abstract] OR "curriculum"[Title/Abstract] OR "knowledge"[Title/Abstract] OR "competence"[Title/Abstract] OR "health education"[MeSH Terms] OR "teaching"[MeSH Terms] OR "learning"[MeSH Terms] OR "education"[MeSH Terms] OR "educational technology"[MeSH Terms] OR "simulation training"[MeSH Terms] OR "high fidelity simulation training"[MeSH Terms] OR "education, graduate"[MeSH Terms] OR "education, professional"[MeSH Terms] OR "health educators"[MeSH Terms] OR "educational personnel"[MeSH Terms] OR "faculty, nursing"[MeSH Terms] OR "education, nursing"[MeSH Terms] OR "education, nursing, graduate"[MeSH Terms] OR "education, nursing, continuing"[MeSH Terms] OR "education, nursing, baccalaureate"[MeSH Terms] OR "education, continuing"[MeSH Terms] OR "knowledge"[MeSH Terms] OR "Curriculum"[MeSH Terms]	410
Scopus	(TITLE-ABS-KEY("nurs*" OR "nursing student*" OR "undergraduate nursing" OR "Students, Nursing") AND	962

	TITLE-ABS-KEY("chatgpt" OR "chat gpt" OR "chat gpt" OR "natural language processing" OR "chatbot*" OR "conversational agent*" OR "artificial intelligent agent*" OR "artificial intelligence agent*" OR "intelligent agent*" OR "AI agent" OR "ai based conversational agent*" OR "intelligent conversational agent*" OR "artificial agent*" OR "virtual assistant*" OR "conversational agent*" OR "ai tutor*" OR "intelligent tutor*" OR "automated tutor*" OR "virtual agent*" OR "intelligent virtual assistant*" OR "generative artificial intelligence") AND TITLE-ABS-KEY("educat*" OR "teach*" OR "training" OR "instruction*" OR "simulat*" OR "interactive learning" OR "gamification" OR "game*" OR "curriculum" OR "knowledge" OR "competence" OR "health education" OR "teaching" OR "learning" OR "education" OR "educational technology" OR "simulation training" OR "high fidelity simulation training" OR "education, graduate" OR "education, professional" OR "health educators" OR "educational personnel" OR "faculty, nursing" OR "education, nursing" OR "education, nursing, graduate" OR "education, nursing, continuing" OR "education, nursing, baccalaureate" OR "education, continuing" OR "knowledge" OR "Curriculum"))	
Web Of Science	“nurs*” OR “nursing student*” OR “undergraduate nursing” OR “Students, Nursing” (Topic) and "chatgpt" OR "chat gpt" OR "chat gpt" OR "natural language processing" OR "chatbot*" OR "conversational agent*" OR "artificial intelligent agent*" OR "artificial intelligence agent*" OR "intelligent agent*" OR "AI agent" OR "ai based conversational agent*" OR "intelligent conversational agent*" OR "artificial agent*" OR "virtual assistant*" OR "conversational agent*" OR "ai tutor*" OR "intelligent tutor*" OR "automated tutor*" OR "virtual agent*" OR "intelligent virtual assistant*" OR “generative artificial intelligence” (Topic) and “educat*” OR “teach*” OR “training” OR “instruction*” OR “simulat*” OR “interactive learning” OR “gamification” OR “game*” OR “curriculum” OR “knowledge” OR “competence” OR “health education” OR “teaching” OR “learning” OR “education” OR “educational technology” OR “simulation training” OR “high fidelity simulation training” OR “education, graduate” OR “education, professional” OR “health educators” OR “educational personnel” OR “faculty, nursing” OR “education, nursing” OR “education, nursing, graduate” OR “education, nursing, continuing” OR “education, nursing, baccalaureate” OR “education, continuing” OR “knowledge” OR “Curriculum” (Topic)	599
BVS/LILACS	((tw:(enfermagem) OR tw:("estudantes de enfermagem") OR tw:("profissionais de enfermagem"))) AND ((tw:(chatbot*) OR tw:("agente conversacional") OR	272

	tw:("assistente virtual") OR tw:("tutor virtual") OR tw:("inteligência artificial") OR tw:("inteligência artificial generativa") OR tw:("modelo de linguagem de grande escala"))) AND ((tw:(educação) OR tw:(ensino) OR tw:(aprendizagem) OR tw:(formação) OR tw:(capacitação) OR tw:(treinamento)))	
CINAHL Complete (via EBSCO)	MeSH descriptor: [Students, Nursing] explode all trees OR MeSH descriptor: [Nursing] explode all trees OR MeSH descriptor: [Nursing Staff] explode all trees OR (nurs*):ti,ab,kw OR (nursing student):ti,ab,kw OR (undergraduate nursing):ti,ab,kw OR (students, nursing):ti,ab,kw OR (students):ti,ab,kw **AND** (chatgpt):ti,ab,kw OR (chat gpt):ti,ab,kw OR (natural language processing):ti,ab,kw OR (chatbot):ti,ab,kw OR (chatbots):ti,ab,kw OR (chat bot):ti,ab,kw OR (conversational agent):ti,ab,kw OR (artificial intelligent agent):ti,ab,kw OR (intelligent agent):ti,ab,kw OR (artificial agent):ti,ab,kw OR (virtual assistant):ti,ab,kw OR MeSH descriptor: [Natural Language Processing] explode all trees OR MeSH descriptor: [Generative Artificial Intelligence] explode all trees **AND** (“education” OR “teach” OR “training” OR “instruction” OR “simulat” OR “interactive learning” OR “gamification” OR “game” OR “curriculum” OR “knowledge”) OR (education):ti,ab,kw OR (teach):ti,ab,kw OR (training):ti,ab,kw OR (instruction):ti,ab,kw OR (simulat):ti,ab,kw OR (interactive learning):ti,ab,kw OR (gamification):ti,ab,kw OR (game):ti,ab,kw OR (curriculum):ti,ab,kw OR (knowledge):ti,ab,kw OR MeSH descriptor: [Health Education] explode all trees OR MeSH descriptor: [Learning] explode all trees OR MeSH descriptor: [Education] explode all trees OR MeSH descriptor: [Educational Technology] explode all trees OR MeSH descriptor: [Education, Graduate] explode all trees OR MeSH descriptor: [Education, Professional] explode all trees OR MeSH descriptor: [Educational Personnel] explode all trees OR MeSH descriptor: [Education, Nursing] explode all trees	511
Cochrane Library	MeSH descriptor: [Students, Nursing] explode all trees OR MeSH descriptor: [Nursing] explode all trees OR MeSH descriptor: [Nursing Staff] explode all trees OR (nurs*):ti,ab,kw OR (nursing student):ti,ab,kw OR (undergraduate nursing):ti,ab,kw OR (students, nursing):ti,ab,kw OR (students):ti,ab,kw **AND** (chatgpt):ti,ab,kw OR (chat gpt):ti,ab,kw OR (natural language processing):ti,ab,kw OR (chatbot):ti,ab,kw OR (chatbots):ti,ab,kw OR (chat bot):ti,ab,kw OR (conversational agent):ti,ab,kw OR (artificial intelligent agent):ti,ab,kw OR (intelligent agent):ti,ab,kw OR (artificial agent):ti,ab,kw OR (virtual assistant):ti,ab,kw	197

	OR MeSH descriptor: [Natural Language Processing] explode all trees OR MeSH descriptor: [Generative Artificial Intelligence] explode all trees **AND** (“education” OR “teach” OR “training” OR “instruction” OR “simulat” OR “interactive learning” OR “gamification” OR “game” OR “curriculum” OR “knowledge”) OR (education):ti,ab,kw OR (teach):ti,ab,kw OR (training):ti,ab,kw OR (instruction):ti,ab,kw OR (simulat):ti,ab,kw OR (interactive learning):ti,ab,kw OR (gamification):ti,ab,kw OR (game):ti,ab,kw OR (curriculum):ti,ab,kw OR (knowledge):ti,ab,kw OR MeSH descriptor: [Health Education] explode all trees OR MeSH descriptor: [Learning] explode all trees OR MeSH descriptor: [Education] explode all trees OR MeSH descriptor: [Educational Technology] explode all trees OR MeSH descriptor: [Education, Graduate] explode all trees OR MeSH descriptor: [Education, Professional] explode all trees OR MeSH descriptor: [Educational Personnel] explode all trees OR MeSH descriptor: [Education, Nursing] explode all trees	
Scielo via Clarivate	“nurs*” OR “nursing student*” OR “undergraduate nursing” OR “Students, Nursing” AND "chatgpt" OR "chat gpt" OR "chat gpt" OR "natural language processing" OR "chatbot*" OR "conversational agent*" OR "artificial intelligent agent*" OR "artificial intelligence agent*" OR "intelligent agent*" OR "AI agent" OR "ai based conversational agent*" OR "intelligent conversational agent*" OR "artificial agent*" OR "virtual assistant*" OR "conversational agent*" OR "ai tutor*" OR "intelligent tutor*" OR "automated tutor*" OR "virtual agent*" OR "intelligent virtual assistant*" OR “generative artificial intelligence” AND “educat*” OR “teach*” OR “training” OR “instruction*” OR “simulat*” OR “interactive learning” OR “gamification” OR “game*” OR “curriculum” OR “knowledge” OR “competence” OR “health education” OR “teaching” OR “learning” OR “education” OR “educational technology” OR “simulation training” OR “high fidelity simulation training” OR “education, graduate” OR “education, professional” OR “health educators” OR “educational personnel” OR “faculty, nursing” OR “OR “education, nursing, graduate” OR “education, nursing, continuing” OR “education, nursing, baccalaureate” OR “education, continuing” OR “knowledge” OR “Curriculum”	6
	**TOTAL**	2957
	**Gray literature**	
OpenAire	“nurs*” OR “nursing student*” OR “undergraduate nursing” OR “Students, Nursing” AND "chatgpt" OR "chat gpt" OR "chat gpt" OR "natural language processing" OR	65

	"chatbot*" OR "conversational agent*" OR "artificial intelligent agent*" OR "artificial intelligence agent*" OR "intelligent agent*" OR "AI agent" OR "ai based conversational agent*" OR "intelligent conversational agent*" OR "artificial agent*" OR "virtual assistant*" OR "conversational agent*" OR "ai tutor*" OR "intelligent tutor*" OR "automated tutor*" OR "virtual agent*" OR "intelligent virtual assistant*" OR “generative artificial intelligence” AND “educat*” OR “teach*” OR “training” OR “instruction*” OR “simulat*” OR “interactive learning” OR “gamification” OR “game*” OR “curriculum” OR “knowledge” OR “competence” OR “health education” OR “teaching” OR “learning” OR “education” OR “educational technology” OR “simulation training” OR “high fidelity simulation training” OR “education, graduate” OR “education, professional” OR “health educators” OR “educational personnel” OR “faculty, nursing” OR “OR “education, nursing, graduate” OR “education, nursing, continuing” OR “education, nursing, baccalaureate” OR “education, continuing” OR “knowledge” OR “Curriculum”	
ProQuest ™ Dissertations & Theses Citation Index (via Clarivate)	“nurs*” OR “nursing student*” OR “undergraduate nursing” OR “Students, Nursing” AND "chatgpt" OR "chat gpt" OR "chat gpt" OR "natural language processing" OR "chatbot*" OR "conversational agent*" OR "artificial intelligent agent*" OR "artificial intelligence agent*" OR "intelligent agent*" OR "AI agent" OR "ai based conversational agent*" OR "intelligent conversational agent*" OR "artificial agent*" OR "virtual assistant*" OR "conversational agent*" OR "ai tutor*" OR "intelligent tutor*" OR "automated tutor*" OR "virtual agent*" OR "intelligent virtual assistant*" OR “generative artificial intelligence”<br>AND “educat*” OR “teach*” OR “training” OR “instruction*” OR “simulat*” OR “interactive learning” OR “gamification” OR “game*” OR “curriculum” OR “knowledge” OR “competence” OR “health education” OR “teaching” OR “learning” OR “education” OR “educational technology” OR “simulation training” OR “high fidelity simulation training” OR “education, graduate” OR “education, professional” OR “health educators” OR “educational personnel” OR “faculty, nursing” OR “education, nursing” OR “education, nursing, graduate” OR “education, nursing, continuing” OR “education, nursing, baccalaureate” OR “education, continuing” OR “knowledge” OR “Curriculum”	37
BDTD CAPES	(enfermagem OR "estudantes de enfermagem" OR "profissionais de enfermagem") AND (chatbot OR "agente conversacional" OR "assistente virtual" OR "tutor virtual" OR "inteligência artificial" OR "inteligência artificial	26

	generativa" OR "modelo de linguagem de grande escala" OR "ChatGPT") AND (educação OR ensino OR aprendizagem OR formação OR capacitação OR treinamento)	
Open Dissertations Via Ebscohost	“nurs*” OR “nursing student*” OR “undergraduate nursing” OR “Students, Nursing” AND "chatgpt" OR "chat gpt" OR "chat gpt" OR "natural language processing" OR "chatbot*" OR "conversational agent*" OR "artificial intelligent agent*" OR "artificial intelligence agent*" OR "intelligent agent*" OR "AI agent" OR "ai based conversational agent*" OR "intelligent conversational agent*" OR "artificial agent*" OR "virtual assistant*" OR "conversational agent*" OR "ai tutor*" OR "intelligent tutor*" OR "automated tutor*" OR "virtual agent*" OR "intelligent virtual assistant*" OR “generative artificial intelligence” AND “educat*” OR “teach*” OR “training” OR “instruction*” OR “simulat*” OR “interactive learning” OR “gamification” OR “game*” OR “curriculum” OR “knowledge” OR “competence” OR “health education” OR “teaching” OR “learning” OR “education” OR “educational technology” OR “simulation training” OR “high fidelity simulation training” OR “education, graduate” OR “education, professional” OR “health educators” OR “educational personnel” OR “faculty, nursing” OR “OR “education, nursing, graduate” OR “education, nursing, continuing” OR “education, nursing, baccalaureate” OR “education, continuing” OR “knowledge” OR “Curriculum”	9
Google Scholar	allintitle: chatbot "nursing" OR "nurse" OR "student" OR "education"	877
	**TOTAL**	1023

*Busca realizada em 13 de outubro de 2025.

Fonte: Elaborado pelos autores, 2025.
