# Supplementary material for: Educational Applications of AI-Based Chatbots in Nursing: A Scoping Review
Source: Nurs Rep. 2026 Mar 3;16(3):87. doi: 10.3390/nursrep16030087 (PMC13028657; doi:10.3390/nursrep16030087)
Supplement: Supplementary file 1 [file nursrep-16-00087-s001.zip › nursrep-4153709-Table S4.pdf]

Table S4 in supplementary material.

Table S4a. Numerical Reconciliation of Study Selection Process

Stage	Databases	Other sources	Total
Records identified	2957	1014	3971
Duplicate records removed	1364	123	1487
Records screened	1593	891	2484
Records excluded	1281	842	2123
Full-text assessed	312	49	361
Full-text excluded	262	33	295
Studies included	50	16	66

Table S4b. Reasons for Full-Text Exclusion (n=295)

Reason for exclusion	n
Not nursing education	24
Not chatbot-based AI	37
Clinical only	2
Review articles	56
Editorial/commentary	16
No empirical data	118
Insufficient methods	42
Total	295
